# Gestational Age–Specific Prevalence of Preeclampsia Among Pregnant Women in Ghana: A Hospital-Based Retrospective Observational Study

**DOI:** 10.1155/bmri/4728838

**Published:** 2025-03-11

**Authors:** Wina Ivy Ofori Boadu, Enoch Odame Anto, Akua Benewaah Asamoah, Ezekiel Ansah, Godfred Yawson Scott, Emmanuel Ekow Korsah, Joseph Frimpong, Valentine Christian Kodzo Tsatsu Tamakloe, Michael Nyantakyi, Agartha Odame Anto, Emmanuel Timmy Donkoh, Kwame Ofori Boadu, Richard Vikpebah Duneeh, Frederick Ayensu, Christian Obirikorang

**Affiliations:** ^1^Department of Medical Diagnostics, Faculty of Allied Health Sciences, College of Health Sciences, Kwame Nkrumah University of Science and Technology, Kumasi, Ghana; ^2^School of Medical and Health Sciences, Edith Cowan University, Perth, Australia; ^3^Centre for Precision Health, ECU Strategic Research Centre, Edith Cowan University, Perth, Australia; ^4^Department of Obstetrics and Gynaecology, Ho Teaching Hospital, Ho, Ghana; ^5^Department of Medical Laboratory Science, Centre for Research in Applied Biology, University of Energy and Natural Resources, Sunyani, Ghana; ^6^Department of Obstetrics and Gynaecology, Kumasi South Regional Hospital, Kumasi, Ghana; ^7^Department of Medical Laboratory Sciences, School of Allied Health Sciences, University of Health and Allied Sciences, Ho, Ghana; ^8^HopeXchange Medical Centre, Kumasi, Ghana; ^9^Department of Molecular Medicine, School of Medical Sciences, College of Health Science, Kwame Nkrumah University of Science and Technology, Kumasi, Ghana

**Keywords:** gestational age, LMICs, maternal outcomes, preeclampsia

## Abstract

**Background:** Preeclampsia is responsible for a significant rate of maternal morbidity and mortality worldwide. Gestational age as a risk factor has a significant impact on fetal outcomes in pregnancies affected by preeclampsia. However, to our knowledge, no study has identified the gestational age–specific prevalence of preeclampsia in Ghana. Thus, this study ascertained the gestational age–specific prevalence of preeclampsia as well as its associated factors.

**Methods:** A hospital-based retrospective observational study was conducted by reviewing data collected from the maternal birth register on 619 pregnant women who delivered at the facility from 1st January 2021 to 31st December 2021.

**Results:** Out of 619 pregnant women, the overall prevalence of preeclampsia among the study participants was 10.5% whereas 80.5% were normotensive pregnant women. The gestational age–specific prevalence of preeclampsia was 2.3%, 2.1%, 4.0%, 1.6%, and 0.5% at < 37 weeks, 37–38 weeks, 39–40 weeks, 41 weeks, and ≥ 42 weeks, respectively. Most of the mothers who had preeclampsia were within the age group of 30–39 years (40, 61.5%), had informal education (41, 64.1%), and were multigravida (40, 61.5%). Age group 30–39 years (aOR = 2.49, 95% CI (1.25–4.96), *p* = 0.0090), C/S (aOR = 2.83, 95% CI (1.46–5.50), *p* = 0.0020), and gestational age category < 37 weeks (aOR = 0.24, 95% CI (0.07–0.78), *p* ≤ 0.0140) and 37–38 weeks (aOR = 0.23, 95% CI (0.08–0.66), *p* = 0.0060) were the independent predictors of preeclampsia, respectively, with head circumference < 33 cm (aOR = 2.09, 95% CI (1.00–4.37), *p* = 0.0490) as the independent complication associated with it.

**Conclusions:** Gestational age–specific prevalence of preeclampsia is high at full-term (39–40 weeks) gestation. Independent risk factors for preeclampsia included maternal age (30–39 years), gestational age (< 37 weeks), and previous caesarean section. Babies of women with preeclampsia are likely to have a small head circumference.

## 1. Background

Preeclampsia (PE), characterized by hypertension and organ damage during pregnancy is a prevalent and severe complication posing risks to both maternal and fetal health, with approximately 2%–5% of pregnancies worldwide being affected [1].

In low- and middle-income countries (LMICs), the prevalence of PE is estimated to range from 1.8% to 16.7%, with the highest rates observed among women of African descent [2]. However, the prevalence is low (2.63%) in high-income countries (HICs) [3], underscoring the disproportionate burden of PE in LMICs. This trend might be due to disparities in healthcare access, diagnostic capabilities, and prenatal care utilization in LMICs. In Ghana, a multicenter prospective cross-sectional study conducted from 2021 to 2022 found the prevalence of PE to be 8.8% [4]. This prevalence underscores the significance of targeted healthcare strategies and interventions to address this relatively high burden of PE and improve maternal outcomes. Also, understanding how Ghana fits into this global trend is essential, as PE remains a significant public health concern in the country.

While there is a comprehensive understanding and routine utilization of clinical presentation, diagnostic criteria, and management strategies for PE, its underlying etiology remains poorly understood; however, a widely accepted perspective attributes PE to abnormal placentation, resulting in significant maternal physiologic dysfunction, characterized by aberrant spiral artery remodeling, placental ischemia, hypoxia, and oxidative stress [5].

Several studies have consistently highlighted advanced maternal age, maternal body mass index (BMI), parity, multiple gestation, a history of diabetes mellitus, pregnancy hypertension, and gestational diabetes as established clinical risk factors for PE [6, 7]. Gestational age emerges as a crucial determinant, with the strongest predictor of fetal mortality and morbidity observed at less than 30 weeks of gestation [8], making it a critical factor influencing clinical management and outcomes in PE.

In Ghana, no study has identified the gestational age–specific prevalence of PE, making it critical to investigate this aspect. There is also a dearth of knowledge on factors associated with PE cases among pregnant women in Ghana. This study is aimed at determining the gestational age–specific prevalence of PE and associated risk factors among pregnant women at HopeXchange Medical Centre, Kumasi, Ghana. These findings hold significant implications for informing decisions and the development of health policies for managing PE and its associated outcomes.

## 2. Methods

### 2.1. Study Design and Setting

This hospital-based retrospective study was conducted using medical data from the maternal birth register containing nulliparous, primiparous, and multiparous women who had singleton pregnancies from 1st January 2021 to 31st December 2021 to determine the gestational age–specific prevalence of PE and associated risk factors among pregnant women at HopeXchange Medical Centre, Kumasi.

HopeXchange Medical Centre is a modern, fully digital center of excellence with a 100-bed capacity. The medical center is one of the supporting hospital facilities to the Komfo Anokye Teaching Hospital (KATH), Ghana's second largest hospital which is situated in the Ashanti Region of Ghana. This affiliation enhances its capacity to provide comprehensive maternal healthcare services in the region. It is situated at Wamase, Kumasi, in the Ashanti Region of Ghana. Wamase is a locality in Ghana. Wamase is situated nearby to the locality of Apere and the town of Aburaso. HopeXchange Medical Centre contains adult and pediatric wards, an intensive care unit, an outpatient center, three state-of-the-art surgical operating rooms, a clinical and research laboratory, a diagnostic and imaging department, an endoscopy unit, an ophthalmic department, and a maternal health unit.

### 2.2. Ethical Consideration

The study was approved by the Committee on Human Research, Publications and Ethics (CHRPE) (CHRPE/AP/621/23), School of Medicine and Dentistry, Kwame Nkrumah University of Science and Technology (KNUST). Written consent was requested from and approved by the Office of Research Support at HopeXchange Medical Centre. Strict measures ensured confidentiality and privacy, with anonymity and securely stored data accessible only to authorized researchers involved in this study.

### 2.3. Study Population and Participant Selection

Hospital records of 619 pregnant women aged 16–40 years who had singleton pregnancies from January 1, 2021, to December 31, 2021, at HopeXchange Medical Centre were obtained using an Excel spreadsheet from the hospital birth registry.

### 2.4. Inclusion Criteria and Exclusion Criteria

This study included all nulliparous, primiparous, and multiparous women who had singleton births at the hospital within the stated period, and all pregnancies at 20 weeks' gestation to term. All nulliparous, primiparous, and multiparous women with gestational age less than 20 weeks, aged less than 15 years (< 15) and above 40 years (> 40), and with twin or multiple gestation were excluded.

### 2.5. Data Collection

Data was collected from the maternal birth register using an Excel spreadsheet which featured:

Sociodemographic variables (maternal age, occupation, and educational level), obstetric parameters (number of antenatal care (ANC) visits, gravidity, parity, gestational age, antepartum hemorrhage (APH), postpartum hemorrhage (PPH), etc.), maternal medical parameters (syphilis, hepatitis B, mother to child HIV transmission, hemoglobin concentration (HB), and intermittent preventive treatment in pregnancy with sulfadoxine–pyrimethamine (IPT/SP) medication), and labour variables (gender, weight, body length, head circumference (HC) and birth abnormality, fetal presentation and fetal lie, caesarean section or delivery through the birth canal, Apgar score, etc.). Missing data were excluded to prevent bias as their inclusion could compromise the study outcome.

PE was defined based on the revised definition by the International Society for the Study of Hypertension in Pregnancy (ISSHP) as a new onset of gestational hypertension (≥ 140 mmHg systolic/90 mmHg diastolic) developed at or after 20 weeks' gestation and with new onset of at least either one of proteinuria (spot check urine protein > 30 mg/mmol (0.3 mg/mg) or > 300 mg/day or at least 1 g/L (“2+” using dipstick testing)) or without proteinuria but the involvements of maternal organ dysfunctions (neurological complications, pulmonary oedema, haematological complications, liver involvement, or acute kidney injury) and or uteroplacental dysfunction [9]. Diagnosis of PE was done by a consultant obstetrician and gynaecologist. Uteroplacental dysfunction was evaluated with ultrasound assessment of fetal growth and umbilical artery Doppler velocimetry or cerebroplacental ratio measurements to assess blood flow redistribution in placental insufficiency according to the International Society for Ultrasound in Obstetrics and Gynaecology guideline [10].

### 2.6. Statistical Analyses

The collected data obtained were entered, coded, edited, and cleaned in Microsoft Excel 2019. All statistical analyses were performed using the Statistical Package for the Social Sciences (SPSS) version 26.0 (Chicago Illinois, United States) and GraphPad Prism version 8.0.1 (GraphPad Software, San Diego California, United States, http://www.graphpad.com). Descriptive statistics were used to present the baseline data variables. Categorical data were presented as frequencies and percentages. The chi-square test/Fisher's exact test and the univariate followed by multivariate logistic regression analysis were employed to test for associations and the strength thereof between the dependent variable (PE) and independent variables. The *p* values less than 0.05 were considered statistically significant for all analyses.

## 3. Results

### 3.1. Sociodemographic and Maternal Obstetric Characteristics of Study Participants

Table 1 shows the demographic characteristics of the study participants. Out of the 619 study participants, 295 (47.7%) were aged 20–29 years, while only 16 (2.6%) were 40 years. Thirteen (2.1%) of the women had no formal education, but the majority had acquired basic education (226, 36.7%), 168 (27.3%) had secondary education, and 209 (33.9%) had tertiary education. Also, 346 (56.7%) of the pregnant women worked in the informal sector, as compared to 197 (32.3%) who were employed in the formal sector. Furthermore, multigravida (372, 60.4%) accounted for the majority of pregnant women, followed by grand multigravida (131, 21.3%) and primigravida (113, 18.3%). The majority of the pregnant women were multiparous (259, 42.0). More than half of the women (536, 96.4%) had ≥ 4 ANC visits (Table 1).

### 3.2. Overall Prevalence of PE Among the Study Participants

**Figure 1:**
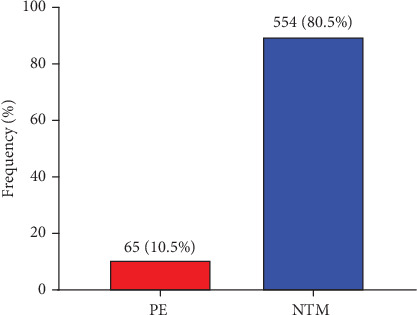
The overall prevalence of preeclampsia among the study participants.

Figure 1 shows the overall prevalence of PE among the study participants. The prevalence of PE among the study participants was 10.5% (65/619) whereas 80.5% (554/619) were normotensive pregnant women (NTM) (Figure 1).

### 3.3. Gestational Age–Specific Prevalence of PE Among the Study Participants

**Figure 2:**
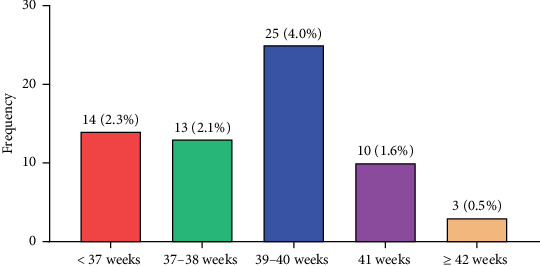
Gestational age–specific prevalence of preeclampsia among the study participants.

The distribution of women according to their gestational age–specific prevalence of PE is shown in Figure 2. The prevalence of PE was 2.3%, 2.1%, 4.0%, 1.6%, and 0.5% at < 37 weeks, 37–38 weeks, 39–40 weeks, 41 weeks, and ≥ 42 weeks, respectively (Figure 2).

### 3.4. Association of Sociodemographic and Maternal Obstetric Characteristics With PE Among Study Participants

Table 2 shows the association of maternal demographic characteristics and obstetric factors with PE among study participants. There was no significant association between participants' level of education (*p* = 0.6830), occupation (*p* = 0.4210), ANC visit (*p* = 0.0930), gravidity (*p* = 0.2350), parity (*p* = 0.2110), anaemia status (*p* = 0.3230), and PE. However, PE was found to be significantly associated with gestational age category (< 37, 37–38, 39–40, ≥ 40, and ≥ 42) (*p* = 0.0001) and age category (*p* = 0.0230) (Table 2).

### 3.5. Association of Adverse Foetomaternal Complications With PE Among the Study Participants

Table 3 shows the association of adverse maternal complications with PE among the study participants. There was no birth asphyxia in a greater percentage (98.5%) of the pregnant women with PE. PE was present in 100% of the mothers, who all gave birth to live babies. There was no significant association between birth asphyxia (*p* = 0.8500), birth abnormalities (*p* = 0.1970), prolonged labour (*p* = 0.1420), PPH (*p* = 0.3640), APH (*p* = 0.3340), obstructed labour (*p* = 0.1420), birth outcome (*p* = 0.4180), and state of placenta (*p* = 0.1420) with PE. This study found a significant association between mode of delivery (*p* = 0.0001) and PE (Table 3).

### 3.6. Association of Labour Characteristics With PE Among the Study Participants

Table 4 shows the association of labour characteristics with PE among the study participants. Both groups (PE and NTM) had a higher percentage of male births (56.5% vs. 56.9%) than female births (43.5% vs. 43.1%). The study found no significant association between baby sex (*p* = 0.9530), full length (*p* = 0.2970), 1-min Apgar score (*p* = 0.8380), and 5-min Apgar score (*p* = 0.5670) with PE. However, a significant association between weight (*p* = 0.0290), HC (*p* = 0.0430), and PE were found (Table 4).

### 3.7. Univariate and Multivariate Logistic Regression Model of Sociodemographic, Obstetric, and Labour Characteristics and Maternal Complication Predictors of PE Among Study Participants

Table 5 shows the maternal sociodemographic, obstetric, and labour characteristics and maternal complications as predictors of PE among the study participants. In a univariate logistic regression model, age category, mode of delivery, fetal gestational age category, weight, and HC were predictors of PE. Women between 30 and 39 years (crude odds ratio (cOR) = 2.39, 95% confidence interval (CI) (1.34–4.24), *p* = 0.0030) were significantly associated with 2.3 times higher risk of being diagnosed with PE when compared with women between 20 and 29 years. Also, women who underwent previous caesarean section (cOR = 3.01, 95% CI (1.73–5.22), *p* = 0.020) were significantly associated with a 3.0 times increased risk of being diagnosed with PE compared with normal delivery. Women with gestational age < 37 weeks (cOR = 0.18, 95% CI (0.07–0.45), *p* ≤ 0.0001), 37–38 weeks (cOR = 0.15, 95% CI (0.06–0.33), *p* ≤ 0.0001), and ≥ 42 weeks (cOR = 0.27, 95% CI (0.10–0.71), *p* = 0.0080) had lower odds of having PE compared with those with a gestational age of 39–40 weeks. Moreover, low baby weight (cOR = 2.69, 95% CI (1.27–5.73), *p* ≤ 0.0100) was significantly associated with 2.6 times of being diagnosed with PE than normal weight. HC < 33 (cOR = 1.84, 95% CI (1.01–3.33), *p* ≤ 0.0450) was significantly associated with 1.8 times the risk of being diagnosed with PE as compared to HC ≥ 33.

After adjusting for the level of education and occupation in a multivariate logistic regression model, having age group 30–39 years (aOR = 2.49, 95% CI (1.25–4.96), *p* = 0.0090), having C/S (aOR = 2.83, 95% CI (1.46–5.50), *p* = 0.0020), and having gestational age category < 37 weeks (aOR = 0.24, 95% CI (0.07–0.78), *p* = 0.0140) and 37–38 weeks (aOR = 0.23, 95% CI (0.08–0.66), *p* = 0.0060) remained significant and were the independent predictors of PE and having HC < 33 (aOR = 2.09, 95% CI (1.00–4.37), *p* = 0.0490) was the independent complication associated with it (Table 5).

## 4. Discussion

This hospital-based retrospective cross-sectional study was carried out at HopeXchange Medical Centre to identify the gestational age–specific prevalence of PE. The overall prevalence of PE in this study was 10.4%, which is higher than the previously reported prevalence of 8.8% in Ghana [4]. However, it is lower than the prevalence reported in Ethiopia (12.3%) [11], and the higher rate of 25.4% is found in another Ghanaian study [12].

We discovered that among pregnant women who gave birth at HopeXchange Medical Centre, the gestational age–specific prevalence of PE was 2.3%, 2.1%, 4.0%, 1.6%, and 0.5% at < 37 weeks, 37–38 weeks, 39–40 weeks, 41 weeks, and ≥ 42 weeks, respectively.

The intricacy of a late-term pregnancy may account for the highest prevalence of PE at 39–40 weeks (4.0%). As the pregnancy progresses, the demands on the placenta and maternal organs increase [13]. The placenta's aging and potential placental insufficiency might contribute to the higher prevalence of PE in this period. Medical intervention, such as labour induction, is more frequent in postterm pregnancies, which may help to reduce the incidence of complications from PE [14, 15]. Pregnancies that have progressed to this point without experiencing any significant problems may also have a lower risk of developing PE as evidenced in a gestational age of ≥ 42 weeks which recorded the least prevalence (0.5%). Also, this might be because some women experiencing PE-related complications might have been induced or delivered by this time, reducing the sample size for this category. The prevalence of PE among a gestational age of 41 weeks was 1.6% which could be because some women experiencing PE-related complications might have been induced or delivered by this time, reducing the sample size for this category. Prevalence of PE was 2.3% and 2.1% for a gestational age of < 37 weeks and 37–38 weeks, respectively, and it could be due to factors such as underlying lifestyle or medical conditions in the mother, genetic predisposition, autoimmune disorders, or problems with the placenta's development, leading to inadequate blood flow to the fetus. Once the highest prevalence was among those in full term and as in term, it has been reported that more than 75% of PE occurs among women with full-term gestation [9]. Also, about one-quarter of PE occurs in preterm gestation [16]. This finding supports this study in that the highest prevalence was found in women with full gestation.

Maternal age is one of the potential risk factors for PE [17]. Women of maternal age 30–39 were 2.49 times more likely to have PE compared to women under 30 in this study. According to a study, among preeclamptic women, adverse pregnancy outcomes were more common in women of advanced maternal age [18]. This may be related to the progressive vascular endothelial damage that occurs with maternal aging and obstruction of maternal spiral arteriolar lumina by atherosis [19]. Furthermore, advanced maternal age increases the risks of poor perinatal outcomes, preterm birth < 37 weeks, IUGR, asphyxia, and perinatal infection in PE pregnant women [17].

Moreover, gestational age < 37 weeks and 37–38 weeks was also associated with a higher risk of PE, according to multivariate analysis. The developing placenta and fetal vasculature may not have fully developed in earlier pregnancies, which increases the risk of the placenta receiving insufficient blood flow (gestational age < 37 weeks and 37–38 weeks) [20, 21]. PE may develop as a result of impaired blood flow [20, 21].

According to this study, having a previous caesarean delivery increased the risk of PE. This is consistent with findings from a study by [4]. Although the exact cause is unknown, it is most likely that the caesarean procedure, which is related to poor placentation, increases the development of PE in subsequent pregnancies [22]. Furthermore, different uterine changes from caesarean delivery may obstruct typical trophoblastic invasion and alter uteroplacental blood flow in subsequent pregnancies, resulting in PE [22].

Also, HC was associated with PE in this study. A study by [23] suggested that fetal HC increases at a greater rate in PE. Increased fetal exposure to neurotrophins, such as brain-derived neurotrophic factor (BDNF), may be the cause of increased fetal HC in PE. Disorders associated with changes in BDNF show abnormalities in brain growth; it is an essential regulator of proper neurodevelopment [24]. Elevated BDNF has also been seen in term PE in the placenta and cord blood [25]. The increased fetal HC in PE may therefore be explained by greater fetal exposure to BDNF at term [23].

## 5. Conclusions

Among pregnant women at HopeXchange Medical Centre, the gestational age–specific prevalence of PE is 2.3%, 2.1%, 4.0%, 1.6%, and 0.5% at < 37 weeks, 37–38 weeks, 39–40 weeks, 41 weeks, and ≥ 42 weeks, respectively. Factors such as maternal age, gestational age, and previous caesarean section are independent risk factors of PE. Babies of women with PE are likely to have a small HC.

### 5.1. Recommendation

Based on the study findings, the following are recommended.

It is recommended that health workers use maternal age, previous caesarean section, and gestational age, as screening tools for the prediction of PE in the study setting as early as possible which will also help manage and prevent complications such as small fetal HC associated with it.

### 5.2. Strengths and Limitations of the Study

The use of gestational age in this study helped to assess the prevalence of PE.

This study has its limitations, even though it provides policymakers with information to help reduce morbidity and mortality from PE. Since secondary data were used, some records about sociodemographic, obstetric, medical, and fetal features were missed.

## Figures and Tables

**Table 1:** Sociodemographic and maternal obstetric characteristics of study participants.

**Variable**	**Frequency (** **n** = 619**)**	**Percentage (%)**
*Age category*		
< 20	22	3.6
20–29	295	47.7
30–39	285	46.1
≥ 40	16	2.6
*Level of education*		
No education	13	2.1
Basic	226	36.7
Secondary	168	27.3
Tertiary	209	33.9
*Occupation*		
Unemployed	67	11.0
Informal	346	56.7
Formal	197	32.3
*Gravidity*		
Primigravida	113	18.3
Multigravida	372	60.4
Grand multigravida	131	21.3
*Parity*		
Nulliparous	210	34.0
Primiparous	148	24.0
Multiparous	617	42.0
*ANC visit (per number of times)*		
< 4	20	3.6
≥ 4	536	96.4

*Note:* Data is presented as frequency (percentage).

Abbreviation: ANC, antenatal care.

**Table 2:** Association of sociodemographic and obstetric characteristics with preeclampsia among study participants.

**Variable**	**Total (** **N** = 619**)**	**NTN (** **N** = 554**)**	**PE (** **N** = 65**)**	**p** ** value**
*Age category*				**0.0230**
< 20	15 (3.2)	12 (3.0)	3 (4.6)	
20–29	224 (48.4)	204 (51.3)	20 (30.8)	
30–39	211 (45.6)	171 (43.0)	40 (61.5)	
≥ 40	13 (2.8)	11 (2.8)	2 (3.1)	
*Level of education*				0.6830
No education	7 (1.5)	6 (1.5)	1 (1.6)	
Basic	172 (37.3)	144 (36.3)	28 (43.8)	
Secondary	122 (26.5)	108 (27.2)	14 (21.9)	
Tertiary	160 (34.7)	139 (35.0)	21 (32.8)	
*Occupation*				0.4210
Unemployed	48 (10.5)	43 (10.9)	5 (7.8)	
Informal	259 (56.7)	218 (55.5)	41 (64.1)	
Formal	150 (32.8)	132 (33.6)	18 (28.1)	
*Gravidity*				0.2350
Primigravida	85 (18.4)	77 (19.3)	8 (12.3)	
Multigravida	285 (61.6)	245 (61.6)	40 (61.5)	
Grand multigravida	93 (20.1)	76 (19.1)	17 (26.2)	
*Parity*				0.2110
Nulliparous	167 (36.0)	144 (36.1)	23 (35.4)	
Primiparous	114 (24.6)	103 (25.8)	11 (16.9)	
Multiparous	183 (39.4)	152 (38.1)	31 (47.7)	
*ANC visit (per number of times)*				0.0930
< 4	14 (3.4)	10 (2.8)	4 (7.1)	
≥ 4	401 (96.6)	349 (97.2)	52 (92.9)	
*Anaemia status*				0.3230
Anaemic	99 (24.8)	89 (25.6)	10 (19.2)	
Nonanaemic	301 (75.3)	259 (74.4)	42 (80.8)	
*Gestational age category (weeks)*				**< 0.0001**
< 37	32 (7.1)	18 (4.7)	14 (21.5)	
37–38	105 (23.4)	92 (24.0)	13 (20.0)	
39–40	247 (55.0)	222 (57.8)	25 (38.5)	
≥ 40	7 (1.6)	4 (1.0)	3 (4.6)	
≥ 42	58 (12.9)	48 (12.5)	10 (15.4)	

*Note:* Data is presented as frequency (percentage); chi-square/Fisher's exact test, *p* value < 0.05 was considered statistically significant for preeclampsia and normotensive mothers. The bold values indicate *p* values which are statistically significant.

Abbreviations: ANC, antenatal care; NTN, normotensive; PE, preeclampsia.

**Table 3:** Association of adverse foetomaternal complications with preeclampsia among study participants.

**Variable**	**Total (** **N** = 619**)**	**NTN (** **N** = 554**)**	**PE (** **N** = 65**)**	**p** ** value**
*Birth asphyxia*				0.8500
No	458 (98.7)	394 (98.7)	64 (98.5)	
Yes	6 (1.3)	5 (1.3)	1 (1.3)	
*Birth abnormalities*				0.1970
No	454 (97.8)	389 (97.5)	65 (100.0)	
Yes	10 (2.2)	10 (2.5)	0 (0.0)	
*Prolonged labour*				0.1420
No	462 (99.6)	398 (99.7)	64 (98.5)	
Yes	2 (0.4)	1 (0.3)	1 (1.5)	
*PPH*				0.3640
No	458 (98.9)	393 (98.7)	65 (100.0)	
Yes	5 (1.1)	5 (1.3)	0 (0.0)	
*APH*				0.3340
No	460 (99.4)	396 (99.5)	64 (98.5)	
Yes	3 (0.6)	2 (0.5)	1 (1.5)	
*Obstructed labour*				0.1420
No	461 (99.6)	397 (99.7)	64 (98.5)	
Yes	2 (0.4)	1 (0.3)	1 (1.5)	
*Birth outcome*				0.4180
Dead	4 (0.9)	4 (1.0)	0 (0.0)	
Alive	460 (99.1)	395 (99.0)	65 (100.0)	
*State of placenta*				0.1420
Incomplete	2 (0.4)	1 (0.3)	1 (1.5)	
Complete	462 (99.6)	398 (99.7)	64 (98.5)	
*Mode of delivery*				**< 0.0001**
Previous C/S	116 (25.4)	87 (22.1)	29 (46.0)	
Normal	341 (74.6)	307 (77.9)	34 (54.0)	

*Note:* Data is presented as frequency (%); Chi-square/Fisher's exact test, *p* value < 0.05 was considered statistically significant for preeclampsia and normotensive mothers. The bold values indicate *p* values which are statistically significant.

Abbreviations: APH, antepartum hemorrhage; C/S, caesarean section; PPH, postpartum hemorrhage.

**Table 4:** Association of labour characteristics with preeclampsia among study participants.

**Variable**	**Total (** **N** = 619**)**	**NTN (** **N** = 554**)**	**PE (** **N** = 65**)**	**p** ** value**
*Fetal sex*				0.9530
Male	262 (56.6)	225 (56.5)	37 (56.9)	
Female	201 (43.4)	173 (43.5)	28 (43.1)	
*Full length (cm)*				0.2970
< 53.0	358 (82.9)	308 (82.1)	50 (87.7)	
≥ 53.0	74 (17,1)	67 (17.9)	7 (12.3)	
*Head circumference (cm)*				**0.0430**
< 33.0	105 (24.4)	85 (22.7)	20 (35.1)	
≥ 33.0	326 (75.6)	289 (77.3)	37 (64.9)	
*Weight (kg)*				**0.0290**
LBW	41 (9.1)	30 (7.7)	11 (18.0)	
Normal	392 (86.7)	345 (88.2)	47 (77.0)	
Macrosomia	19 (4.2)	16 (4.1)	3 (4.9)	
*1-min Apgar score*				0.8380
Low	10 (2.2)	9 (2.3)	1 (1.6)	
Intermediate	57 (12.3)	48 (12.0)	9 (14.3)	
Normal	395 (85.5)	342 (85.7)	53 (84.1)	
*5-min Apgar score*				0.5670
Low	5 (1.1)	5 (1.3)	0 (0.0)	
Intermediate	10 (2.2)	8 (2.0)	2 (3.2)	
Normal	447 (96.8)	386 (96.7)	61 (96.8)	

*Note:* Data is presented as frequency (percentage); chi-square/Fisher's exact test, *p* value < 0.05 was considered statistically significant for preeclampsia and normotensive mothers. The bold values indicate *p* values which are statistically significant.

Abbreviation: min, minutes.

**Table 5:** Univariate and multivariate logistic regression model of sociodemographic, obstetric, and labour characteristics and maternal complication as predictors of preeclampsia among study participants.

**Variable**	**cOR (95% CI)**	**p** ** value**	**aOR (95% CI)**	**p** ** value**
*Age category (years)*				
20–29	1.00		1.00	
< 20	2.55 (0.66–9.80)	0.1730	1.57 (0.20–12.7)	0.6650
30–39	2.39 (1.34–4.24)	0.0030	2.49 (1.25–4.96)	**0.0090**
≥ 40	1.86 (0.38–8.96)	0.4420	3.36 (0.62 -18.19)	0.1600
*Level of education*				
Tertiary	1.00		1.00	
No education	1.10 (0.13–9.63)	0.9290		—
Basic	1.29 (0.70–2.37)	0.4190		—
Secondary	0.86 (0.42–1.77)	0.6770		—
*Occupation*				
Formal	1.00		1.00	
Unemployed	0.85 (0.30–2.43)	0.7660		—
Informal	1.38 (0.76–2.50)	0.2890		—
*Mode of delivery*				
Normal	1.00		1.00	
Previous C/S	3.01 (1.73–5.22)	< 0.0001	2.83 (1.46–5.50)	**0.0020**
*Gestational age category*				
39–40	1.00		1.00	
< 37	0.18 (0.07–0.45)	< 0.0001	0.24 (0.07–0.75)	**0.0140**
37–38	0.15 (0.06–0.33)	< 0.0001	0.23 (0.08–0.66)	**0.0060**
≥ 40	0.96 (0.19–5.03)	0.9660	0.98 (0.14–6.95)	0.9840
≥ 42	0.27 (0.10–0.71)	0.0080	0.45 (0.14–1.50)	0.1950
*Weight*				
Normal	1.00		1.00	
Low	2.69 (1.27–5.73)	0.0100	1.24 (0.41–3.78)	0.7000
Macrosomia	1.38 (0.39–4.90)	0.6220	1.04 (0.25–4.20)	0.9610
*Head circumference*				
≥ 33	1.00		1.00	
< 33	1.84 (1.01–3.33)	0.0450	2.09 (1.00–4.37)	**0.0490**

*Note:* Data is presented as odds ratio (95% confidence interval). Binary logistic regression analysis was performed to obtain odds ratios. *p* value < 0.05 was considered statistically significant. 1.00, reference. The bold values indicate *p* values which are statistically significant.

Abbreviations: aOR, adjusted odds ratio, CI, confidence interval; cOR, crude odds ratio; inf, infinity.

## Data Availability

The datasets used and/or analyzed during the current study are available from the corresponding author upon reasonable request.

## Nomenclature

PE: preeclampsia
LMICs: low- and middle-income countries
BMI: body mass index
ANC: antenatal care
APH: antepartum hemorrhage
BDNF: brain-derived neurotrophic factor
PPH: postpartum hemorrhage
HB: hemoglobin concentration
IPT/SP: intermittent preventive treatment in pregnancy with sulfadoxine–pyrimethamine
HC: head circumference
NTN: normotensive
ISSHP: International Society for the Study of Hypertension in Pregnancy
SPSS: Statistical Package for the Social Sciences
CHRPE: Committee on Human Research, Publications and Ethics
HIV: human immunodeficiency virus
